# Rebamipide gargle and benzydamine gargle in prevention and management of chemo-radiotherapy and radiotherapy-induced oral mucositis in head and neck cancer patients (randomized clinical trial)

**DOI:** 10.1186/s12903-024-04379-3

**Published:** 2024-06-01

**Authors:** Basma Elsaadany, Samah M. Anayb, Karim Mashhour, Mohammed Yossif, Fat’heya Zahran

**Affiliations:** 1 Department of Oral Medicine and Periodontology, Faculty of Dentistry, Cairo University, Cairo, Egypt; 2 Clinical Oncology, Kasr El Ainy- Cairo University, Cairo, Egypt; 3 Egyptian drug authority, Cairo, Egypt

**Keywords:** Rebamipide, Benzydamine HCl, Oral mucositis, Radiotherapy, Chemoradiotherapy, Head & neck cancer

## Abstract

**Objectives:**

This study aimed to evaluate the preventive and therapeutic effects of rebamipide gargle in comparison with benzydamine in head and neck cancer patients undergoing radiotherapy with or without chemotherapy.

**Materials and methods:**

Phase III randomized clinical trial was conducted from January 2021 till August 2022 on one hundred patients with head and neck cancer receiving high doses of radiotherapy. These patients were equally allocated into either rebamipide group or benzydamine group, The measured outcomes were the incidence of oral mucositis ≥ grade1, according to the WHO mucositis scale, in addition to the duration, and the onset of oral mucositis.

**Results:**

There was no statistically significant difference between the two groups, regarding the incidence of a severe grade of oral mucositis (WHO grades 3), as well as the onset and duration of oral mucositis. Both gargles succeeded to prevent the development of WHO grade 4 oral mucositis. Side effects reported were mainly burning sensation in benzydamine group and nausea in rebamipide group.

**Conclusion:**

Rebamipide mouthwash was as beneficial as benzydamine mouthwash in minimizing the incidence of severe oral mucositis induced by treatment of head and neck cancer. However, rebamipide gargle proved to be superior to benzydamine in terms of reduction in the severity of the radiation-induced oral mucositis.

**Trial registration:**

The trial was registered in the protocol Registration and Result system of Clinical Trials (Registration ID: NCT04685395)0.28-12-2020.

## Background

“Head and neck cancer” (HNC ) refers to a diverse range of cancers, including those of the oral cavity, nasal cavity, salivary glands, oropharynx, hypopharynx, larynx, nasopharynx, and paranasal sinuses [1]. Overall, in the U.S. in 2019, there were 53,000 new cases and 10,860 deaths from head and neck cancer [2]. In 2030, the World Health Organization predicts that 439,000 cases of mouth and oropharynx cancer would be reported [3]. The current range of HNC treatment options includes surgery, radiation, chemotherapy, targeted therapy, and immunotherapy, which is typically administered in combination [4]. Radiotherapy and surgery are the most effective treatments for head and neck cancer.

Oral mucositis (OM) is one of the most harmful and toxic side effects of cancer treatment. According to reports. It affects 20–40% of individuals receiving conventional chemotherapy and 75–85% of those receiving bone marrow transplants [5]. Because of the proximity of the oral cavity to the areas involved in head and neck cancer, the incidence in patients receiving radiation for these conditions might reach 100% [6]. The symptoms, which can vary in severity, start after cumulative exposure to 15 Gy and become more pronounced if the total dose reaches 60 Gy [7]. Aside from the impairment of oral health, OM is also accompanied by difficulty chewing, swallowing, eating, and drinking due to pain and inflammation of the esophagus and oral mucosa. If the debilitating symptoms are left untreated, they may cause decreased appetite, which may impair nutrition. If they are severe enough, it may cause treatment to be discontinued [8]. Therefore, it is crucial that mucositis be avoided wherever feasible, or at the very least, treated to lessen its severity and any side effects. While there is some evidence to support the use of numerous therapies for the treatment or prevention of mucositis, no gold standard medication for the prevention or treatment of OM is currently available [9].

The evidence recommended and suggested some interventions [9], however, in the real life the management of mucositis mainly depends on institutional and/or personal levels [10].

Benzydamine hydrochloride is a non-steroidal anti-inflammatory drug, that has also demonstrated topical anesthetic, analgesic, and antibacterial properties and is used to treat inflammatory disorders such as radiotherapy-induced oral mucositis (RIOM) or chemotherapy-induced oral mucositis (CIOM) [11, 12]. The International Society of Oral Oncology and the Multinational Association of Supportive Care in Cancer recently recommended it in the MASCC/ISOO guidelines as one of the most essential drugs for preventing RIOM [13].

Rebamipide has been used effectively to treat gastric disorders [14] and used for the management of numerous oral diseases as recurrent aphthous ulceration & Behcet disease [15]. Several trials were conducted to evaluate the efficacy of rebamipide in the management of oral mucositis versus placebo [16, 17]. According to Akagi and his colleagues’ meta-analysis in 2019, gargling with rebamipide is more effective than a placebo for treating oral mucositis in patients receiving chemo-radiotherapy, especially for severe cases of Grade 3 or above. However, due to insufficient evidence, there could be no recommendations regarding rebamipide.

Accordingly, this study aimed to evaluate the preventive and therapeutic effects of rebamipide gargle in comparison with benzydamine HCl in head and neck cancer patients undergoing radiotherapy alone or concomitant with chemotherapy.

## Subjects and methods

### Study design

The present study is a phase III, randomized controlled, clinical trial, with parallel groups in a single center, 100 Participants diagnosed as having head and neck cancer (HNC) and receiving radiotherapy only or chemoradiotherapy were recruited from January 2021 to August 2022, and randomly allocated into one of two groups with an allocation ratio of 1:1.

### Eligibility criteria

#### Inclusion criteria

Eligible patients were: (i) male and female patients with HNC, (ii) prescribed radiotherapy of at least 60 Gy [18], (iii) willing to participate, (iv) able to rinse, (v) older than 18 years of age, (vi) having normal renal and liver functions and (vii) with unstimulated salivary flow rate more than 0.1 ml/min (excluding salivary hypofunction) [19] before the start of radiotherapy, (viii) non-smokers or quitted smoking during radiotherapy.

#### Exclusion criteria

(i) Patients having any source of infection in the oral cavity, (ii) Patients using dentures, (iii) patients having previous history of allergy to rebamipide or benzydamine hydrochloride, (iv) Patients who had prior cancer treatment.

### Study setting

The participants were recruited from Kasr El-Einy Center of Radiation Oncology and Nuclear Medicine, Faculty of Medicine, Cairo University.

Pre-treatment measures: A signed informed consent was obtained from each patient after explaining the steps of the study and discussing the treatment plan in addition to oral hygiene instructions.

Then, basic demographics and clinical data including age, gender, type of malignancy, site to be treated, if taking concomitant chemotherapy, dose of radiotherapy, and any history of allergy to drugs were recorded in prepared sheets.

### Randomization and concealing

Enrolled patients were randomly distributed (simple randomization) into 2 groups using an online randomization program: http://www.Randome.org.

In accordance with the random sequence acquired by the computer software, numbers were written on folded opaque sheets and placed in opaque sealed envelopes. Before beginning any procedure, all those documents were prepared. The numbers were placed in envelopes, and the patients were free to choose their own numbers.

The treatment was supplied to the patient by the pharmacist labelled as treatment A or B, according to the random sequence.

The benzydamine HCl and rebamipide mouthwashes were identical in colour and bottle (benzydamine HCl mouthwash was emptied in containers identical to the prepared rebamipide gargle containers by the pharmacist).

The study participants, main investigator (responsible for recruiting, examination and follow up) and the statistician were blind to the treatment entity.

### Ethical considerations

The study protocol was developed regarding good medical practice standards in accordance with the Declaration of Helsinki and was revised and approved by the ethical committee of the Faculty of Dentistry, Cairo University. Approval No: (191,113). Date of approval: 26th November, 2019. The aim of the study was explained to all subjects participating in this study and written informed consents were obtained before enrolment.

### Treatment preparation and application

#### Control group

Patients were blindly allocated to receive commercially available benzydamine HCl (0.15%) gargle.

#### Intervention group

Patients were blindly allocated to receive a rebamipide gargle which was prepared by an expert pharmacist according to a standardized method; the formula was administered as a mouthwash in a similar amount, dosage form, and duration as in the control group.

The patients in both groups were provided with measuring cups (graduated 5–15 ml) and they were instructed to use 5 ml gargle. The gargle was given to each patient to cover 1-week use (250 ml). All bottles of gargle in both groups were returned every week for quality and compliance control.

Patients were instructed to rinse or gargle with 5 mL every 3 h (6 times daily) and the gargle should generally be used undiluted, only swish for 30 s to 1 min and spit it, no swallowing. In addition, avoid eating, drinking, or rinsing with water for at least 10 min after using the gargle. This was performed a day before the first day of RT to the end of the treatment.

Patients also were instructed that in case of developing any allergic reaction (in case of absence of history of sensitivity or first exposure), they should stop the treatment and report the situation to the main investigator. Also, they instructed to report by phone changing the ability of eating different food consistencies (liquid, hard) as an indication of changing the mucositis grade if occur in between the assessment sessions.

### Preparation of gargle

The rebamipide solution was prepared by the method of Hanawa et al. [20]. To prepare a volume of 100 mL of Rebamipide gargle solution, the following were included: 100 mg of Rebamipide (Meisei Chemical, Japan), 1 g of polyethylene oxide (Sigma Aldrich Chemie GmbH - Switzerland), and 0.4 g of Carrageenan (Kerry Group - Ireland), with the addition of 6 mL of 4% lidocaine (Sigma Aldrich Chemie GmbH - Switzerland). The mixture was mixed gently. This mixture was added to 5 mL of Glycerol (El-Nasr Pharmaceutical Chemical Company - Egypt) and ultrasonicated for 3 min. The flavoring and coloring agents were dissolved in deionized water (Arab Company for Pharmaceutical and Medicinal plants MEPACO-MEDIFOOD Egypt) and this solution was used to complete the final volume to 100 mL.

### Outcomes assessment

Primary outcome was to compare the incidence of oral mucositis by performing weakly intraoral clinical examination (OM was reported in case we detected ≥ grade1 lesion according to the WHO scale) [21] after the use of rebamipide and benzydamine HCl gargles in head and neck cancer patients undergoing radiotherapy or chemoradiotherapy.

Secondary outcomes included comparing the therapeutic effect of rebamipide gargle and benzydamine HCl in head and neck cancer patients undergoing radiotherapy with or without chemotherapy in terms of : (i) The onset of oral mucositis (by recording the day when symptoms of oral mucositis started to develop from the beginning of radiotherapy). (ii) The duration of OM (by recording duration, in days from the onset of oral mucositis to complete healing). (iii) The degree of OM severity (reported according to WHO scale after weekly intraoral clinical examination) (iv) reporting any detected side effects of the mouthwashes.

*WHO Mucositis scale* [22] was used for the assessment of the degree and severity of mucositis affecting oral intake of food. The score ranges from 0 to 4 which includes:

*Grade 0*: No changes, Grade 1: Soreness / (+) erythema, *Grade 2*: Erythema (++), ulcer, can eat hard food, *Grade 3*: Ulcer, (+++) erythema with ulcers that necessitate liquid food, *Grade 4*: Ulcers with hemorrhage and necrosis, alimentation not possible.

### Sample size

A power analysis was performed to calculate the minimum sample size required to accept the outcome of a statistical test, with the confidence level α = 0.050. So, the power level was 0.80. The primary outcome is incidence of OM thus we used the incidence reported for two comparison group in study done by Sahebjamee et al. [23] where incidence of OM of grade 2 in test group 30.8% and in control group 7.7%. The estimated sample size was 45 patients in each group, after correction 50 patients in each group. We used OpenEpi, Version 3 to calculate the sample size.

### Statistical methods

The results were analyzed using SPSS version 28. Qualitative data was described using numbers and percentages. Relation between qualitative data was done using the Chi-square test or Fisher’s exact test as appropriate, while quantitative data was described as mean and standard deviation (SD) or median (range). The Kolmogorov– Smirnov single-sample test was used to determine the data’s normality. The onset and duration of oral mucositis were not normally distributed. To compare the two groups, the Mann-Whitney test was used. *P*-value less than 0.05 was considered statistically significant.

## Results

**Fig. 1:**
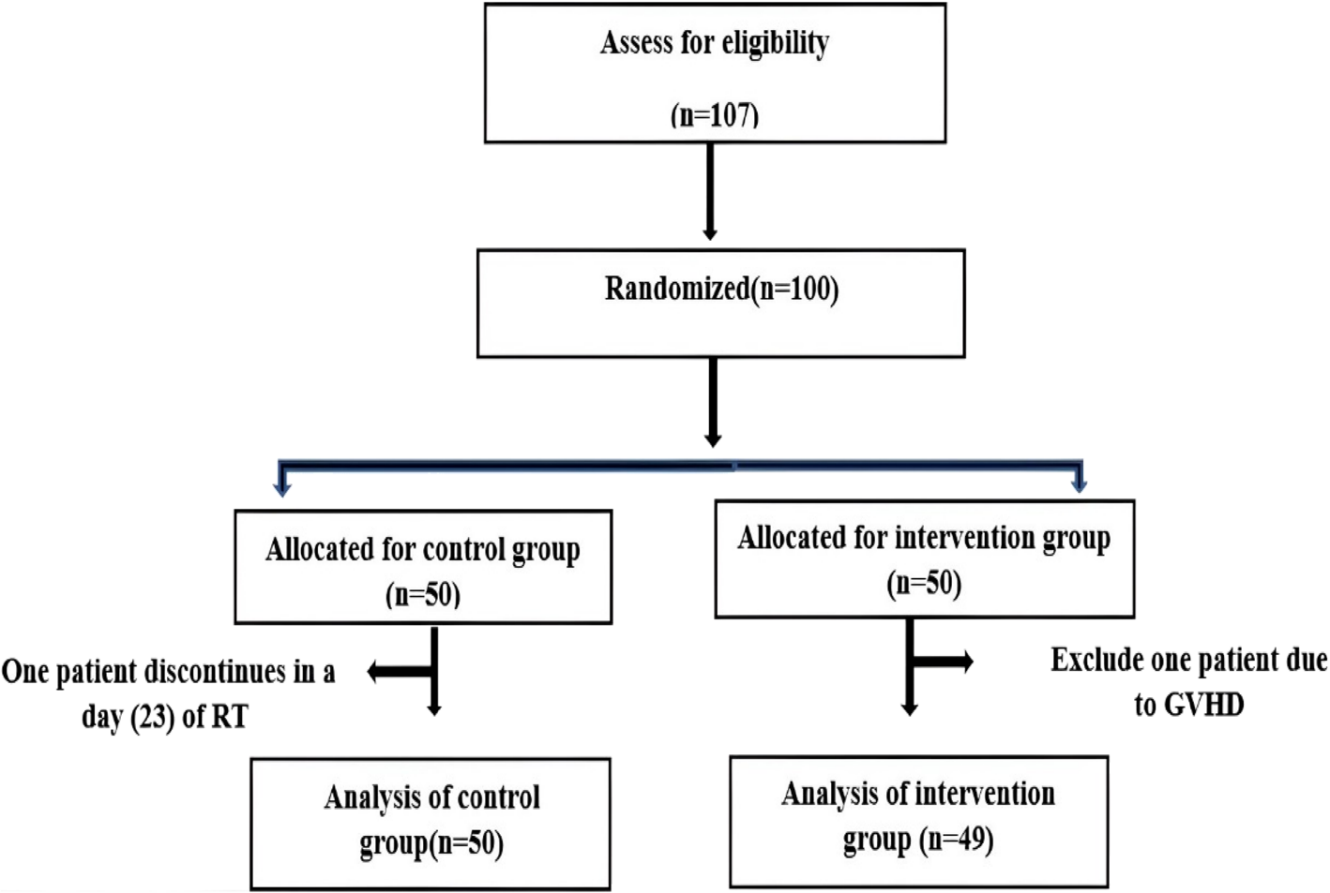
Flow chart of participants enrollment and analysis

The study was conducted in the period of 20 months from January 2021 to August 2022. Although 50 patients were recruited in each of the two groups, one patient in the benzydamine group developed intolerable oral burning on using the gargle and discontinued it at week 5, and one patient in the rebamipide group was excluded due to the appearance of oral graft versus host disease at week 4 (Fig. 1).

### Base line characteristics of the patients

There was no statistically significant difference between the two study groups regarding age, sex, type of cancer, site of cancer, the technique of radiotherapy, radiation dose, and chemotherapy (all patients included were 50 in each group) (Table 1).

**Table 1:** Patients’ demographic data & disease characteristics

Characteristic	Control group (*n* = 50)	Intervention group (*n* = 50)	*p*-value
**Age (years)**			
Mean ± SD	55.9 ± 14.1	52.7 ± 12.8	0.180
Median (range )	59 (21–79)	55 (25–75)	
**Gender**			
Female	13 (26%)	14 (28%)	0.822
Male	37 (74%)	36 (72%)	
**Diagnosis**			
Squamous cell carcinoma	25 (50%)	34 (68%)	0.181
Salivary gland cancer^a^	6 (12%)	5 (10%)	
Others^a^	19 (38%)	11 (22%)	
**Site**			
Parotid	4 (8%)	2 (4%)	0.393
Oral cavity	10 (20%)	8(16%)	
Larynx	15 (30%)	21 (42%)	
Cervical lymph node &submandibular S.G	2 (4%)	5 (10%)	
Others^a^	19 (38%)	14 (28%)	
**Radiotherapy technique**			
3D Conformal	4 (8%)	2 (4.0%)	0.687
IMRT	46 (92%)	48 (96.0%)	
**Radiotherapy dose**			
60 Gy	36 (72.0%)	35 (70.0%)	0.967
66 Gy	5 (10%)	5 (10%)	
70 Gy	9 (18.0%)	10 (20.0%)	
**Chemotherapy**			
No	29 (58.0%)	23 (46.0%)	0.230
Yes	21 (42.0%)	27 (54.0%)	

^a^Other diagnosis includes( lymphoma, hemangioma, Neuroblastoma Medulloblastoma)

Salivary gland cancer^a^ includes (Xpleomorphic adenoma, adenoid cystic carcinoma, and mucoepidermoid carcinoma), and other^a^ sites include (the middle ear, nasal cavity, maxillary sinus, and external auditory meatus )

### Oral mucositis incidence

Only 37 patients out of 100 patients in the study developed mucositis making the incidence of mucositis in the whole sample 37%, out of them 27 (66.7%) mucositis patients were having chemoradiotherapy while only 10 (33.3%) patients of mucositis cases occurred in patients having radiotherapy only.

**Fig. 2:**
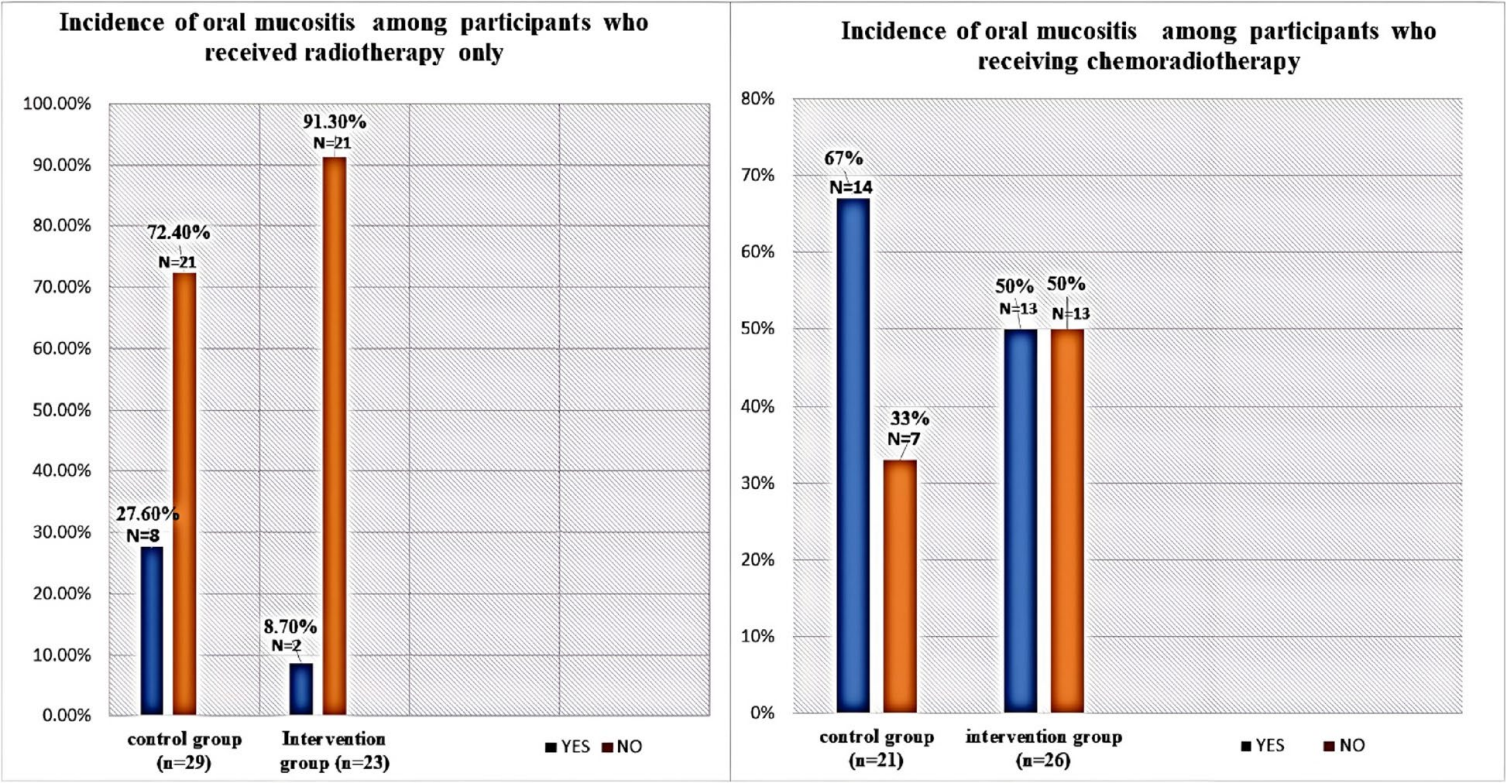
Bar chart showing the incidence of oral mucositis in participants who received radiotherapy with or without chemotherapy

Despite the lower incidence of oral mucositis detected in the rebamipide group, there was no statistically significant difference in incidence of OM between the two studied groups (*p* = 0.196) (Table 2). Similarly, no statistically significant difference was found between the two study groups in the subgroup analysis comparing the incidence of OM in patients taking chemoradiotherapy or radiotherapy only (p-0.374 and 0.086, respectively) (Fig. 2).

**Table 2:** Incidence of oral mucositis in the study groups as total and as differentiated into chemoradiotherapy and radiotherapy subgroups

Incidence of oral mucositis	Benzydamine group	Rebamipide group	Total	*p*-value
Chemoradiotherapy	14 (51.8%)	13 (48.2%)	27 (100%)	0.374
Radiotherapy	8 (80%)	2 (20%)	10 (100%)	0.086
All participants	22 (59.4%)	15 (40.6%)	37 (100%)	0.196

### Oral mucositis onset and duration

There was no statistically significant difference between the two groups regarding onset or duration of oral mucositis as explained in (Table 3).

**Table 3:** Comparison of onset and duration of oral mucositis between study groups

Characteristic	Benzydamine HCl (*n* = 50) (median/range)	Rebamipide (*n* = 49) (median/range)	*p*-value
The onset of oral mucositis (days)	25.5 (10–32)	25 (13–33)	**0.951**
Duration of oral mucositis (days)	16 (7–63)	15 (7–56)	**0.936**

### Maximum WHO grades of OM

Comparing the frequency of severity grades of mucositis between the two groups revealed that there was a statistically significant increase in frequency of grade 1 mucositis in rebamipide group compared to benzydamine HCl group (*p*-value = 0.009). While no statistically significant differences were detected between the two groups in frequency of grade 2 or grade 3 (*p*-value = 0.15, *p*-value = 0.43, respectively), as shown in (Table 4).

**Table 4:** Maximum grade of oral mucositis between the study groups

Maximum Oral Mucositis Grade	Benzydamine HCl Group (*n* = 50)	Rebamipide group(*n* = 49)	*p*-value
Grade 1	2 (9.1%)	7 (46.7%)	0.009
Grade 2	14 (63.6%)	6 (40%)	0.157
Grade 3	6 (27.3%)	2 (13.3%)	0.431

**Fig. 3:**
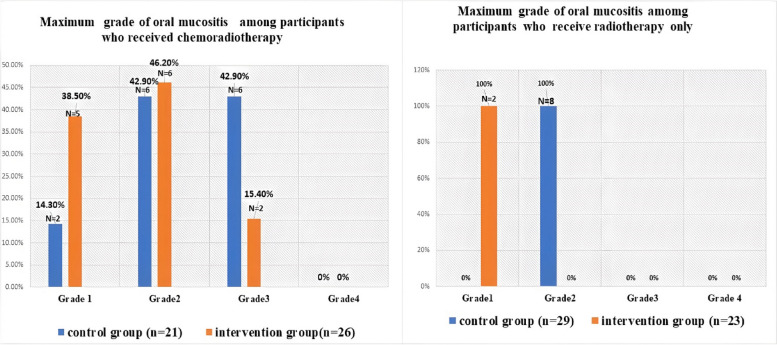
Bar chart showing a maximum grade of oral mucositis among the participants who received radiotherapy with or without chemotherapy

Subgroup analysis of oral mucositis severity in relation to combination of chemoradiotherapy or radiotherapy alone revealed that all the grade 3 mucositis cases were detected in combined therapy in both study groups with no statistically significant difference in number of cases between the study groups. While in radiotherapy only in rebamipide group all cases with mucositis were grade 1, while in benzydamine HCl group all mucositis cases were grade 2 (Fig. 3).

### Oral mucositis resolution among study participants

#### Complete resolution at the end of radiotherapy

More than three-fourths of the patients (76.2%) in the control group achieved complete resolution of oral mucositis at the end of radiotherapy compared to 60% in the intervention group with no statistically significant differences (*p*-value = 0.456).

#### Side effects reported by patients

20% of the participants in the control group complained of burning sensation compared to 4.1% in the intervention group with a statistically significant difference (*p*-value = 0.015). While, 28.6% in the rebamipide group complained of nausea compared to 0% in the control group with statistically significant difference (*p*-value < 0.001).

## Discussion

Oral mucositis (OM) remains among the most unbearable complications of radiotherapy or chemoradiotherapy for head and neck cancer (HNC) patients. It causes painful ulcers and erythema, problems in eating, drinking, and speech, weight loss, and treatment interruptions in addition to having an impact on the quality of life. Several agents and medications have been utilized in this condition’s prophylaxis, therapy, or pain alleviation [24]. Unfortunately, up till now, and over the years, there has been minimal improvement in its prevention or treatment. There is no approved medication for prevention or definitive treatment for the condition [9]. Thus, a great variety in the daily practice still exists as described in the recent study by Bergamaschi et al. who conducted a survey to highlight the clinical practice in management of OM in 25 Italian Centres [10].

Through previous studies, the incidence of radiation-induced mucositis was estimated to be 40–80% in the literature [25]. Another retrospective study conducted by Martin et al. [26] showed that the incidence of oral mucositis in patients with head and neck tumors ranged from 22.9 to 89.7% according to the site, where laryngeal cancer had a risk of 48.4%, nasopharyngeal 22.8%, tongue 89.7%, and when considering the oral cavity in general, it was 61%. In our study, the incidence of oral mucositis in all study participants was 37%. As the highest percentage of patients involved in the present study had laryngeal primary sites, the incidence thus could be considered in accordance with the previously mentioned studies. In our study, about 48% of the patients received chemotherapy concomitantly with radiotherapy. The incidence of OM was found to be increased in those patients (66.7%), while 33.3% only (10 patients) of mucositis cases occurred in radiotherapy only as previously noted by other authors [27].

In the present study, despite the lack of significant difference in the incidence of oral mucositis in both study groups, there was a significant difference in the distribution of OM grades (*p* = 0.044). The number of patients with grade 1 mucositis was significantly higher in the rebamipide group as compared to the benzydamine HCl group, in addition to lower incidence of grades 2,3 in rebamipide group compared to benzydamine HCl group, which emphasize the efficacy of rebamipide in reducing the severity of oral mucositis. Similarly, in the meta-analysis conducted by Akagi et al. [28] they reported that rebamipide gargling reduced the incidence of grade 3 oral mucositis.

The efficacy of rebamipide gargle in reducing the severity of oral mucositis and delaying the onset of oral mucositis in patients undergoing chemoradiation was reported in a study conducted by Yasuda et al. [17], where only 4 cases from 12 (33.3%) developed grade 3 OM in rebamipide group in comparison to the placebo group, where 10 cases from 12 (83.3%) developed grade 3 severity. An important remark in the present study is that no grade 4 mucositis was identified in either group and which was in accordance with the study conducted by Sahebjamee et al. [23] who compared benzydamine HCl to the Alovera mouth wash. The results showed that both interventions were able to prevent the development of mucositis grade 4. Also, this finding is consistent with Yasuda et al. [17], where in the rebamipide group there was only one case that developed grade 4 mucositis versus 4 cases in the placebo group. Rebamipide has anti-inflammatory properties and inhibits the production of pro-inflammatory cytokines. Moreover, rebamipide can increase the capacity of the epithelial barrier and decrease the transit of macromolecules over this barrier. It also demonstrates immunoregulatory capabilities, which can control lymphocyte proliferation and cytokine release [29].

Our study also reveals that benzydamine HCl is beneficial in the prevention of oral mucositis induced by high-dose radiotherapy, where the patients in our study received at least 60 Gy. Also, it showed a significant effect in patients receiving chemoradiation. Benzydamine HCl’s effectiveness in high-dose radiation with or without chemotherapy was also investigated by Rastogi et al. [30]. In contrast to our study, they did not demonstrate any benefits from the preventive rinse with benzydamine HCl in the group of patients undergoing chemoradiation. The difference between the mentioned study and ours could be due to different patients’ inclusion criteria. Only 18% o our study’s participants had the oral cavity as their major site, compared to 57% i the previously mentioned study. Another difference was the radiotherapy method used in their study, which was 100% 3D CRT, while in our trial 3D CRT was used in only 6%, nd 94% had IMRT. IMRT has been claimed to decrease the incidence of severe mucositis, xerostomia, dysphagia, weight loss of the patients, need for nasogastric tubing and it improved the treatment-compliance compared to 3D CRT in locally advanced HNC patients treated by chemo-radiotherapy [31]. All these variations could explain the difference in OM incidence in the two studies.

The expert panel (MASCC/ISOO) agreed to recommend the use of benzydamine HCl mouth rinse for the prevention of OM in patients treated for HNC with moderate RT doses (up to 50 Gy) without chemotherapy [32]. Another systematic review, which was released in 2019, looked at anti-inflammatory drugs used to treat and prevent OM in patients receiving Radiotherapy, chemotherapy, chemoradiotherapy, and hematopoietic stem cell transplantation. The panel (MASCC/ISOO) concluded that benzydamine HCl mouthwash was the only intervention with level II evidence supporting its ability to prevent RT-CT-induced OM [9, 13]. The anti-inflammatory activity of benzydamine HCl has been linked to its ability to inhibit the release of pro-inflammatory cytokines (TNFα, IL-1, and MCP-1), without affecting other inflammatory cytokines (IL-6, IL-8), and, critically, anti-inflammatory cytokines (IL-10, IL-1ra), unlike other NSAIDs, which act by inhibiting prostaglandin synthesis [33].

Regarding the therapeutic efficacy in this study, results showed a slight decrease in duration in favor of rebamipide, however, the difference was statistically non-significant. The mean of OM duration in the control group was 16 versus 15 days in the rebamipide group regarding recovery or resolution from oral mucositis. In both groups, most cases got resolved at the end of radiotherapy. It should be noted that in both groups the mucositis duration was higher in patients receiving chemoradiation than those receiving radiation only.

The study had some limitations and should be interpreted considering its weaknesses. First, the study was performed at a single center and included a small number of patients with oral cancer as we targeted patients with various types of head and neck cancers, which could have a negative effect on the results. As it has been reported that the site of cancer has a crucial impact on the occurrence of OM [34]. In addition, further statistical subgroup analysis to correlate gargle administration and other variables could not be performed due to the need for greater sample sizes. Another limitation was the variability of patients in terms of whether they received chemotherapy or not and the duration of treatment if 6 or 7 weeks. This might have resulted in heterogeneity. For future research, we recommend conducting studies with larger sample sizes to allow for subgroup analysis in patients with head and neck cancer. Also, we recommend evaluation of higher doses of rebamipide and different routes of administration whether local or systemic as mouth rinse covers the oral cavity only but doesn’t cover the pharyngeal area that could be the actual source of patients’ complaints.

## Conclusion

Both rebamipide and benzydamine are almost equally effective for the treatment of radiation-induced oral mucositis and have potential preventive effects on the development of oral mucositis in head and neck cancer patients receiving high-dose radiotherapy or chemoradiation therapy. However, rebamipide gargle seems superior to benzydamine HCl in terms of reduction in the severity of the radiation-induced oral mucositis but the combination of chemotherapy with radiotherapy seems to make the prevention and management of oral mucositis equally difficult for both rebamipide and benzydamine.

## Data Availability

The datasets used and/or analyses used during the current study are available from the corresponding author on reasonable request.
